# Subjective safety and self-confidence in prehospital trauma care and learning progress after trauma-courses: part of the prospective longitudinal mixed-methods EPPTC-trial

**DOI:** 10.1186/s13049-017-0426-5

**Published:** 2017-08-14

**Authors:** David Häske, Stefan K. Beckers, Marzellus Hofmann, Rolf Lefering, Paul A. Grützner, Ulrich Stöckle, Vassilios Papathanassiou, Matthias Münzberg

**Affiliations:** 1 Faculty of Medicine, Eberhard Karls University Tübingen, 72076 Tuebingen, Germany; 2 Department of Anesthesiology, University Hospital RWTH, Aachen, 52074 Aachen, Germany; 3 Emergency Medical Service, Fire Department, City of Aachen, 52057 Aachen, Germany; 4 Faculty of Health, University of Witten/Herdecke, 58448 Witten, Germany; 5 Institute for Research in Operative Medicine, University of Witten/Herdecke, 51109 Cologne, Germany; 6 Department of Trauma Surgery and Orthopedics, BG Hospital Ludwigshafen, BG Trauma Center Ludwigshafen, Ludwig-Guttmann-Str. 13, D-67071 Ludwigshafen, Germany; 7 Department of Traumatology and Reconstructive Surgery, BG Hospital Tuebingen, 72076 Tuebingen, Germany; 8 Institute of Medical Psychology, University Hospital of the Saarland and Medical Faculty of the University of Saarland, /Saar, 66421 Homburg, Germany

**Keywords:** Safety, Allied health personnel, Trauma care, Competence, Learning progress, Self-confidence, Skill, Structure

## Abstract

**Background:**

Prehospital trauma care is stressful and requires multi-professional teamwork. A decrease in the number of accident victims ultimately affects the routine and skills and underlines the importance of effective training. Standardized courses, like PHTLS, are established for health care professionals to improve the prehospital care of trauma patients. The aim of the study was to investigate the subjective safety in prehospital trauma care and learning progress by paramedics in a longitudinal analysis.

**Methods:**

This was a prospective intervention trial and part of the mixed-method longitudinal EPPTC-trial, evaluating subjective and objective changes among participants and real patient care as a result of PHTLS courses. Participants were evaluated with pre/post questionnaires as well as one year after the course.

**Results:**

We included 236 datasets. In the pre/post comparison, an increased performance could be observed in nearly all cases. The result shows that the expectations of the participants of the course were fully met even after one year (*p* = 0.002). The subjective safety in trauma care is significantly better even one year after the course (*p* < 0.001). Regression analysis showed that (ABCDE)-structure is decisive (*p* = 0.036) as well as safety in rare and common skills (both *p* < 0.001). Most skills are also rated better after one year. Knowledge and specific safety are assessed as worse after one year.

**Conclusion:**

The courses meet the expectations of the participants and increase the subjective safety in the prehospital care of trauma patients. ABCDE-structure and safety in skills are crucial. In the short term, both safety in skills and knowledge can be increased, but the courses do not have the power to maintain knowledge and specific subjective safety issues over a year.

**Trial registration:**

German Clinical Trials Register, ID DRKS00004713, registered 14. February 2014

**Electronic supplementary material:**

The online version of this article (doi:10.1186/s13049-017-0426-5) contains supplementary material, which is available to authorized users.

## Background

Emergency care professionals are faced with stressful and complex situations in prehospital care of seriously injured patients [1–3]. Especially in sophisticated, complex and possible rare situations, stress can be detected with multiple stress measurements by heart rate and salivary cortisol measurements as well as workflow analysis, both in reality and in simulation training [1, 3]. Moorthy et al. showed in surgical settings that stress causes more skill and knowledge-based errors [4]. However, in medical simulation training, it was demonstrated - by means of salivary alpha-amylase analysis - that training caused similar stress to real clinical situations. On the other hand, stress was reduced in the post-test and performance was improved [5].

Today, lower incidence of severely injured trauma patients, therefore decreased routine and considerable stress on health care providers underline the importance of effective training in emergency medicine [6].

In the 1970s the treatment of trauma patients in the emergency room became more standardized by the introduction of Advanced Trauma Life Support (ATLS), which provided a new structure in the care for severely injured patients [7]. An associated prehospital equivalent to ATLS is the Pre-Hospital Trauma Life Support (PHTLS) concept. PHTLS is a worldwide established concept with two-day courses for medical providers with the aim to improve the prehospital care of trauma patients.

In the Emergency Medical Service (EMS)-district Wiesbaden (Germany) a previous training concept has been revised due to lack of learning success and satisfaction of participants. At the instigation of the medical director, PHTLS courses were mandatorily established for all paramedics in the EMS Wiesbaden [8].

### Goals of this investigation

Under the circumstances that a large EMS- district introduces this standard training, the goal of this study is to investigate the subjective safety in prehospital trauma care and learning progress by paramedics in a longitudinal analysis. Special attention is given to the longitudinal safety and assessment, not on a short-term increase.

## Methods

### Study design

This was a prospective pre-post intervention trial and was part of the mix-method longitudinal EPPTC (Effect of Paramedic Training on Prehospital Trauma Care)-study evaluating the subjective and objective changes in participants and real patient care through the courses. The complete study is described in the previously published study protocol [9].

### Study setting and selection of participants

The study was performed in the EMS in Wiesbaden (Germany). The operational district in Wiesbaden has five commissioned EMS agencies (four charities, one private provider).

In the context of various difficulties and problems, the controlling authority committed all paramedics to attend the PHTLS courses to create uniform structures and principles [8].

### Intervention

The two-day PHTLS courses are a worldwide standard for paramedics and emergency physicians with the aim to improve prehospital care for trauma patients. PHTLS courses are characterized by a large variety in the teaching methods (e.g. lectures, practical case studies, skill training), with a close instructor-participant ratio (1:4), many practice activities and continuous interaction. In addition to various skills, the priority-based structure ABCDE (Airway, Breathing, Circulation, Disability and Exposure), is taught and practiced in scenario-based training sessions. Teachings correspond with the key recommendations of the German Guideline on Treatment of Patients with Severe and Multiple Injuries [10].

### Data collection and processing

The course participants were interviewed with a questionnaire concerning their level of knowledge, skills and safety in prehospital trauma care. This data was collected at three time points: at the beginning of the course (before the first lesson: t0 “pre”), at the end of the course (before the course-results were presented: t1 “post”) and as well as one year after the course (t2 “after”). The circumstances of the data collection were identical. The questionnaires were pseudonymized with a four-digit code to represent the relationship between the different times.

### Questionnaire development

The questionnaires were developed by an interdisciplinary team consisting of medical educators, emergency physicians, sociologists and psychologists. The questionnaire development was based on unstructured literature research and focus groups interviews of participants from previous courses, as well as on the experience of the expert panel.

Questions should include the subjective safety in skills, but also knowledge and decision making. Moreover, the question was how satisfied the participants were with the training program.

We used single-item scales in the questionnaire, which were constructed based on practical experience and the envisaged training. For that we used numerical endpoint named scales with a 7-point likert scale to avoid ceiling or floor effects [11]. The range of the scales for participants was from −3 (strongly disagree) to +3 (totally agree), including 0. For statistical calculation, we transformed the scale to 1 to 7.

The core set of questions to evaluate the intervention was asked at three time points. Additionally, there are some questions which were asked only for the first-time point t0 and questions which were asked only after a year.

### Primary data analysis

The sample size calculation for the questionnaires with a power of 85% for an effect size d = 0.2 resulted in 238 needed questionnaires in each group. A two-tailed *p*-value <0.05 was considered statistically significant. As data was not normally distributed and because of loss to follow-up, we added 10%, finally *n* = 262 questionnaires per group.

### Statistical analysis

We assessed the construct validity by means of exploratory factor analysis: Bartlett’s test of sphericity and the Kaiser-Meyer-Olkin measure of sampling adequacy were used to check for the appropriateness of the factor analysis. We ran a principal component analysis (PCA) with varimax rotation. Eigenvalues greater than 1.0 were required to retain component factors, and factor loadings of 0.5 or greater were considered satisfactory for the interpretation of the factor structure.

Internal consistency reliability was determined using Cronbach’s alpha coefficient. Values ≥0.70 are acceptable [12].

Because data was not normally distributed the Wilcoxon signed-rank test was used for paired continuous variables and the χ^2^-Test or fisher’s exact test for categorical variables. A two-tailed *p*-value <0.05 was considered statistically significant. For correlations with ordinal-scaled data, Spearman’s rank correlation coefficient was calculated. A linear regression was performed to identify predictors with relevant impact on the main question. Durbin-Watson was checked for autocorrelation of the predictors, and the residuals for normal distribution. Regression coefficients are given with standard error and the respective *p*-value of the model. All data was analysed using the statistical software SPSS (Version 24.0, IBM Inc., Armonk, NY, USA). For continuous variables, data is shown as mean ± standard deviation, as well as median. For categorical variables, percentages are presented.

## Results

In principle, we received 312 questionnaire sets. Overall 55 cases were excluded because of a missing time point t2. We started with 236 sets and performed a separate non-responder analysis. Between the intervention and the follow up 21 students had further trauma training, so they were excluded as well.

### Students characteristics

Demographic characteristics of the surveyed participants is shown in Table 1. The age of the participants and the professional experience correlate as expected (*r* = 0.84, *p* < 0.001).

**Table 1:** Demographic characteristics of the students

	n	%
Age, years		
mean + SD	36.1 ± 10.2	
Min	20	
Max	63	
not reported	*n* = 27	
Sex		
Male	146	61.9
not reported	5	2.3
Professional experience, years		
0–2	44	18.5
3–4	22	9.4
5–6	20	8.3
7–8	18	7.5
9–10	18	7.5
11–12	17	7.1
13–14	22	9.4
≥ 15	76	32.3

### Non-responder analysis

The non-responder (*n* = 55, 17.6%) implied less women than the responder (21.8% versus 36.7%, *p* = 0.041). Both the mean age (35.8 versus 36.1 years, *p* = 0.852) and the professional experience (*p* = 0.985) showed no differences between the two groups. To see if non-responder were particularly dissatisfied with the course, the item "my expectations for the course have been met" was tested for both groups. The mean for the non-responders was 5.0 points and for responders 6.25 points (*p* = 0.180).

### Principal component analysis

The Kaiser-Meyer-Olkin analysis yielded an index of 0.847, and Bartlett’s test of sphericity gave χ^2^ = 1798.262 (*p* < 0.0001); these indicate the appropriateness of the data for PCA. Four factors with eigenvalues ≥1.0 were extracted by PCA and accounted for 64.1% of the overall variance. As shown in Table 2, the first factor (denoted as expectations) accounted for 30.6% of the total variance, and it included 5 items with factor loadings between 0.62–0.89. The second factor (common procedures) accounted for 19.6% of the variance with factor loadings between 0.53–0.81. The third factor (preparation and literary usage) accounted for 7.3% of the variance, comprised just two items with factor loadings 0.66–0.79. The fourth factor (rare procedures) accounted for 6.6% of the variance with factor loadings between 0.55–0.87. The factor loading of one question was only 0.45 and could not be assigned to one of the four factors. The naming of the factors was determined by the factual context and literature [13, 14].

**Table 2:** Factors with description

Factors	Description	Number of items	Cronbach’s alpha	Item means
Factor 1	expectations	5	0.885	6.05
Factor 2	common procedures	5	0.837	5.15
Factor 3	preparation, literature	2	0.672	5.12
Factor 4	rare procedures	3	0.601	3.75

### Expectation and preparation

Expectations are presented in accordance with the factor analysis as shown in Table 3. Based on the median, four items were evaluated consistently in factor 1 equal by 6.0.

**Table 3:** Results

	QUESTION	TIME POINTS			DIFFERENCES		
		pre t0 MW ± SDmedian	post t1 MW ± SDmedian	after t2 MW ± SDmedian	t0-t2 *p*-value	t0-t1 *p*-value	t1-t2 *p*-value
EXPECTAITONS	1. I have high expectations for the course/my expectations have been fulfilled.	5.6 ± 1.2 6.0	6.2 ± 0.9 6.0	5.9 ± 1.1 6.0	0.002	<0.001	<0.001
	2. I expect/have an increased safety in the assessment of the kinematics.	5.9 ± 1.1 6.0	6.1 ± 1.0 6.0	5.6 ± 1.3 6.0	0.009	0.190	<0.001
	3. I expect/have more safety in the classification of critical/non-critical patients.	6.1 ± 1.0 6.0	6.3 ± 0.7 6.0	5.8 ± 1.2 6.0	0.010	0.006	<0.001
	4. I expect to/I can treat life-threatening situations faster.	6.2 ± 0.9 6.0	6.2 ± 0.8 6.0	5.7 ± 1.3 6.0	<0.001	0.517	<0.001
	5. I expect/could to expand my knowledge in trauma care.	6.4 ± 0.9 7.0	6.5 ± 0.7 7.0	6.1 ± 1.1 6.0	0.003	0.371	<0.001
COMMON PROCEDURES	1. I feel safe in airway management	5.1 ± 1.0 5.0	5.9 ± 0.8 6.0	5.4 ± 1.2 5.0	0.001	<0.001	<0.001
	2. I feel safe in proper handling with neck collars	5.7 ± 0.9 6.0	6.4 ± 0.7 6.0	6.3 ± 0.9 7.0	<0.001	<0.001	0.977
	3. I feel safe in removing a helmet	5.2 ± 1.0 5.0	5.8 ± 0.9 6.0	5.9 ± 1.2 6.0	<0.001	<0.001	0.125
	4. I feel safe in the rescue off the vehicle (extrication)	4.8 ± 1.0 5.0	5.8 ± 0.8 6.0	3.8 ± 1.9 4.0	<0.001	<0.001	<0.001
	5. I feel safe in treatment of traumatological emergencies	4.8 ± 1.0 5.0	5.9 ± 0.7 6.0	5.7 ± 0.8 6.0	<0.001	<0.001	<0.001
RARE PROCEDURES	1. I feel safe in thoracic needle decompression	2.2 ± 1.5 1.0	4.5 ± 1.5 5.0	2.8 ± 1.7 2.0	<0.001	<0.001	<0.001
	2. I feel safe in the proper handling of the spineboard	5.2 ± 1.1 5.0	6.1 ± 1.0 6.0	5.8 ± 1.1 6.0	<0.001	<0.001	0.008
	3. I feel safe in the proper handling of the pelvic sling	3.9 ± 1.6 4.0	6.0 ± 0.9 6.0	5.6 ± 1.1 6.0	<0.001	<0.001	<0.001

A *p*-value of <0.05 is considered statistically significant

The last item “I expect/could to expand my knowledge in trauma care.” got the highest expectation value with median 7.0, which was fulfilled in the post measurement with median 7.0. After one year, the value fell on median 6.0.

Apart from the median, mean values showed a partly significant increase from t0 to t1. After a year, when comparing t1 to t2, a significant fall could be seen. In comparison of t0 to t2, all items decreased significantly, except for the expectations for the course, which had exceeded in post values (*p* < 0.001) and also after one year (*p* = 0.002).

Factor 3 “Literature and course preparation” included the item “I am anxious regularly to do further studies by medical journals.” and was evaluated just before the course (mean 5.3 ± 1.1). The item “By the course manual I feel well prepared/has prepared me well for the course” was evaluated at all three time points. Time point t0 was 4.9 ± 1.3, t1 was 5.2 ± 1.2 and t2 4.9 ± 1.4. The difference between before the course to one year after is not significant (*p* = 0.95). The difference from before the course to directly after the course is significant (*p* = 0.012). The rating of t1 to t2 is also significant (*p* = 0.014).

### Common procedures

Common procedures are also shown in Table 3. Handling neck collars and removing helmets are the only skills that don’t drop significantly from t1 to t2. All common procedures are significantly better rated after one year, with exception of the extrication procedure. One of the most important requests to the course is the safety in the treatment of trauma care. This was assessed by the participants as significantly better after the course, even after a year (*p* = 0.001).

### Rare procedures

The thoracic needle decompression was obviously the skill with the least safety and suspected routine of all skills before the course. Even here the classification after one year was significantly better than prior to the course; nonetheless, Table 3 shows the biggest changes. The pelvic sling offered the largest learning effect directly after the course and was assessed as the spine board to be significantly safer in handling even one year after than before the course.

### Additional questions

Additional questions are shown in Additional file 1. If the single item “I attend the kinematics more than before the course” (5.4 ± 1.4) is divided in two groups by its median (≥6), one year after the course (t2), it shows that provider who pay more attention to kinematics are safer in the assessment if it (*p* < 0.001).

Providers who tend to use the ABCDE structure for patient assessment (item “I use the ABCDE structure in the care of trauma patients”, 6.0 ± 1.2) divided by is median (≥6)) stated that they are better in classification of critical or non-critical patients (*p* < 0.001). Also, the calculation of the use of the ABCDE-structure and safety to treat life-threatening situations faster, shows a moderate correlation *r* = 0.598, *p* < 0.001.

The willingness to learn or for further education, measured by the participation in other courses, is equally distributed throughout all ages (*p* = 0.35). Participants who have attended additional courses, stated that they frequently educate themselves by reading journals etc. (*p* = 0.095).

The regression analysis showed that subjective safety in treatment of traumatological emergencies after one year was significantly influenced using the ABCDE-structure for patient assessment (*p* = 0.036), and as a surrogate marker for common skills the handling neck collars (*p* < 0.001) and for rare skills the thoracic needle decompression (*p* < 0.001), as shown in Table 4.

**Table 4:** Linear regression with one major question as a dependent variable at time point t2

Dependent variable: I feel safe in treatment of traumatological emergencies			
Predictor	Coefficient (SE)	95% CI	*p*-value
Work experience	0.00 (0.02)	−0.03 – 0.03	0.859
I use the ABCDE-structure in prehospital trauma care	0.09 (0.04)	0.01–0.18	0.036
I feel safe in thoracic needle decompression	0.10 (0.03)	0.05–0.15	<0.001
I feel safe in proper handling with neck collars	0.43 (0.05)	0.33–0.53	<0.001
After one year, my expectations have been fulfilled	0.05 (0.05)	−0.04 – 0.15	0.275
I am anxious regularly to do further studies by medical journals.	0.03 (0.04)	−0.04 – 0.10	0.404
Sex	−0.07 (0.09)	−0.25 – 0.10	0.400

A *p*-value of <0.05 is considered statistically significant

## Discussion

**Fig. 1:**
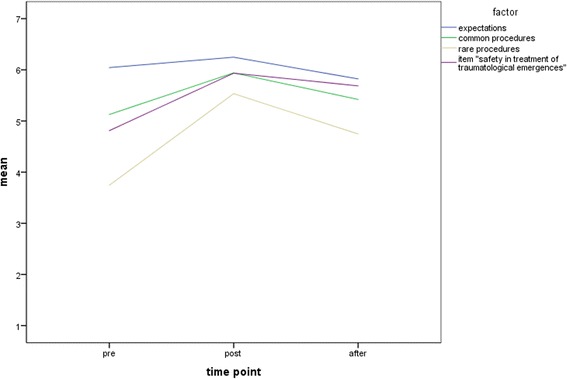
The figure shows the summarized mean values according to the factors, as well as the single major issue “safety in treatment of traumatological emergencies”. The x-axis shows the three time points

The aim of prehospital trauma courses is to gain the assurance in the traumatological skills by improving the knowledge of trauma care, to be able to act faster in life-threatening situations. Cognitive knowledge, technical skills and clinical judgment are the main pillars for healthcare providers [15]. The EPPTC-Trial investigates the impact of such courses and has shown that the trainings improve documentation quality, which was used as a surrogate endpoint for learning effectiveness and awareness [16]. It was demonstrated that participants used certain parts of training in real patient care, thereby suggested that the learning methods of prehospital trauma training are effective. The current study part used questionnaire survey to identify subjective safety. The results show that expectations for the course were exceeded after one year (*p* = 0.002). However, expectations for knowledge and specific questions to safety were met as expected or increased rated after the course, but significantly lower after one year than before the course. Skills, especially rare skills, were mostly significantly better. Figure 1 shows the means of the factors. As described in Table 1, the medians are stable, but Fig. 1 shows the different development of the mean values over the time points.

### Expectations

The medians in the expectation-group show a steady value of 6, even after one year. The expectation for “expand my knowledge” had the highest value before the course (7) and is also met after the course, but dropped after one year to median 6. This might be due to the fact that the course had not enough power to retain knowledge over one year. That knowledge quickly evaporates is not unknown [17]. On the other hand, Mohammad et al. showed that knowledge and skills in the related ATLS courses are increased first, but then declined after half a year, without knowing whether Mohammad et al. have determined this as subjective or objective parameters. The present data showed this change only in the knowledge. The problem of knowledge verification by pre/post-test has already been discussed [16].

However, the course increases the safety in a direct pre/post-comparison, but individual assessments on safety aspects regarding kinematics, classification and speed decrease after one year. A short-term effect in direct pre/post comparison is also described in other studies [18].

Interestingly, nearly all mean values dropped from t0 to t2 significantly, but only with small differences. By contrast the expectations were exceeded significantly after one year (*p* < 0.001). This is remarkable, because participants were told to attend the course and did not participate at their request and it is known that compulsory lessons are sometimes worse evaluated than voluntary events [19].

By the participants, the major issue “safety in the treatment of traumatological emergencies” was in the pre/post comparison as well as after one year significantly higher rated than before the course (both *p* < 0.001). This question is the major issue and is highlighted as single item in Fig. 1.

### Preparation

The value for preparation by course manual is from t0 and t2 not different (*p* = 0.95). T1 is significantly higher evaluated as t0 and t2, possibly because it was noticed that in post-test the questions can be solved with the knowledge of the manual. Münzberg et al. showed that the participants of ATLS courses had best evaluated the skills and scenarios [20]. Most German participants in medical courses prefer practical trainings to theoretical knowledge. Because the manual had 648 pages, perhaps a narrower manual would be recommended as well as new technologies (e.g. mobile apps).

### Common procedures

All common skills were assessed better after one year than before the course, except for safety in the extrication procedure. A reason could be that extrication procedures may be rare in urban emergency services, and the trained procedure of Rapid Extrication must probably be constantly trained to be fit. Therefore, it is interesting that this procedure was assessed as a common procedure, as well as airway management. For German paramedics, invasive airway management skills are certainly not a regular procedure; however, this skill may be well-trained with supraglottic devices in the context of regularly resuscitation training. Nevertheless, the assessment of respiratory management is even better after one year.

As the use of the cervical collar is a regular skill, the more astonishing is it that PHTLS courses improve the safety in this skill right after the course, as well as after a year (*p* < 0.001). In the period, up to one year, own training could also lead to improvement. However, this still appears to be important because the correct application of the cervical collar is often faulty [21].

### Rare procedures

Overall rare procedures show the greatest changes. The safety of using the spineboard is significantly better after one year than before the course (*p* < 0.001), possibly also by own training or application during this time. Although the estimate t2 is on mean lower than t1 (*p* = 0.001), the median is still the same as directly after the course. The fact that the spineboard slips to the rare procedures can be explained in the factor analysis: the difference from eigenvalue for rare procedures (0.551) to common procedures (0.545) is low. It is to be assumed that further analyses shift the spineboard to common procedures.

Thoracic needle decompression is with an incidence around 1.1% extremely rare [14]. Thus, the initial uncertainty in this measure is not surprising. The rating in this study showed an extreme rise and fall immediately after the course and a year after. The value is the lowest after a year overall, but it is even better after one year than before the course (*p* < 0.001). However, the results of safety in this skill are widely varying.

### Major issue

In further question one year after the course, participants agree subjectively to an improvement in patient care after the training. A similar result is also found in a Swiss study in which 85% of the participants see advantages after the introduction of PHTLS [22].

To detect influencing factors concerning our lead issue and major question, safety in treatment of traumatological emergencies, the regression analysis shows that a structure in assessment and treatment is essential for subjective safety, as well as safety in skills and procedures. Work experience or therefore age does not matter, which means that in this study inexperienced paramedics feel equal safe as experienced paramedics – after the training. This is interesting, because young professionals are usually in greater temporal proximity to their up-to-date school-based or university-based knowledge. With increasing experience, the experience will be of greater importance than the systematic knowledge base [23]. The ideal learning psychological approach is the ability to form illness scripts of pattern recognition, which is learned with increasing experience and to combine this with strategies for solving problems supported by e.g. checklists or treatment structure [24]. But also the willingness of these participants to learn and to participate in further training was equally distributed through all ages.

That means that subjective safety in prehospital trauma care depends on structure (ABCDE) and well -trained skills and procedures, independent of age or work experience. The PHTLS-courses use the well-known ABCDE-structure, but presumably this can be transmitted to any assessment/treatment structure.

### Challenges and limitations

This study-part focuses on subjective evaluation and cannot clarify to what extent self-assessment and actual competence match. It is known that self-assessments are not reliable to assess quality medical treatment [25]. For the assessment of students’ capabilities in emergency situations there are further assessments discussed, which differ from ordinary OSCEs [26]. The professional experience also correlates differently with the actual experience, in the sense of patient contacts and corresponding measures [27]. Kreinest et al. showed that the correct application of cervical collars and self-assessment therefore were diametrically divergent [21]. In this context, it seems important to point out that the present study deliberately investigated items for subjective assessment. In order to reconcile self-assessment and reality, it is important to provide feedback for the participants. Consistent feedback structure is an important part of the PHTLS courses. Because hundreds of paramedics cannot be trained by the same instructor team, we have discussed this influence in the study protocol [9]. Because the course regulations for that courses have a high standard in internal quality assurance and a well-structured instructor manual, we assume that there is no relevant or just minimal influence. Matching subjective with objective measurement is investigated in other study parts of the EPPTC-Trial [9].

Whether safety or assessment dropped, or whether the participants have become more critical, cannot be finally clarified with the present questionnaire. It was also discussed, to which extend expectations of subjective safety must be met. The participants had high expectations before the course, which must be achieved. This may be a weakness of the questionnaire or an imprecise question. Subjective safety and confidence are closely intertwined, without being able to separate them further [28]. However, the increased subjective safety should be discussed in context of reduced knowledge and specific safety after one year. If this leads to the fact that the participants now have furthermore confidence, without an objective basis for it, it would be fatal for the patients. This must be clarified in the video analyses as part of our trial [9].

In statistical analysis, we saw, especially in the expectation-group, significant differences between the time points, with small differences between the mean values but stable medians. But the difference in the consideration and result of mean versus median in this method is obviously not only a discussion point for us [29].

Even if skills or procedures in this context are understood as the craftiness of the hand, the questionnaire cannot clarify whether the participants understand the manual implementation or the associated knowledge regarding the indication, contraindication, etc. in the case of questions about skills.

## Conclusion

The result shows that the expectations of the participants in the course were met even after one year. In the pre/post comparison, an increased evaluation is possible almost all subjects. The subjective safety in trauma care is significantly better even one year after the course. Decisive are (ABCDE)-structure and safety in skills. Most skills are also rated better after one year. Knowledge and specific safety are assessed worse after one year.

## Additional file

Additional file 1: Rotated component matrix. (DOCX 28 kb)

## Abbreviations

ATLS: Advanced trauma life support
EMS: Emergency Medical Service
PHTLS: Pre-Hospital trauma life support
