# Supplementary material for: Subjective safety and self-confidence in prehospital trauma care and learning progress after trauma-courses: part of the prospective longitudinal mixed-methods EPPTC-trial
Source: Scand J Trauma Resusc Emerg Med. 2017 Aug 14;25:79. doi: 10.1186/s13049-017-0426-5 (PMC5557465; doi:10.1186/s13049-017-0426-5)
Supplement: Additional file 1: — Rotated component matrix. (DOCX 28 kb) [file 13049_2017_426_MOESM1_ESM.docx]

**Additional file**

**Rotated Component Matrix**

| **Rotated Component Matrix** | | | | |
| --- | --- | --- | --- | --- |
|  | Component | | | |
|  | 1 | 2 | 3 | 4 |
| by the book, I feel well prepared for the course. | ,244 | ,176 | ,661 | ,184 |
| I have high expectations for the course. | ,622 | ,237 | ,333 | -,045 |
| I expect an increased safety in the assessment of the kinematics. | ,886 | -,036 | ,059 | ,060 |
| I expect more safety in the classification of critical / non-critical patients. | ,888 | -,033 | ,073 | ,073 |
| I expect to treat life-threatening situations faster. | ,868 | -,004 | ,072 | ,025 |
| I expect to expand my knowledge in trauma care. | ,828 | ,124 | ,138 | -,052 |
| I feel safe in thoracic needle decompression. | -,168 | ,129 | ,467 | ,558 |
| I feel safe in airway management. | ,086 | ,533 | ,459 | ,113 |
| I feel safe in the proper handling of the spineboard. | ,147 | ,545 | ,026 | ,551 |
| I feel safe in the proper handling of the pelvic sling. | ,061 | ,195 | -,010 | ,867 |
| I feel safe in proper handling with neck collars. | ,166 | ,684 | ,218 | ,155 |
| I feel safe in treatment of traumatological emergencies. | -,050 | ,752 | ,308 | ,121 |
| I feel safe in removing a helmet. | ,042 | ,809 | ,110 | ,100 |
| I feel safe in the rescue off the vehicle (extrication). | ,086 | ,754 | ,114 | ,159 |
| # t0 Ich bin bestrebt, mich regelmäßig durch medizinische Fachzeitschriften fortzubilden. | ,205 | ,182 | ,786 | -,073 |
| Extraction Method: Principal Component Analysis. Rotation Method: Varimax with Kaiser Normalization. | | | | |
|  | | | | |


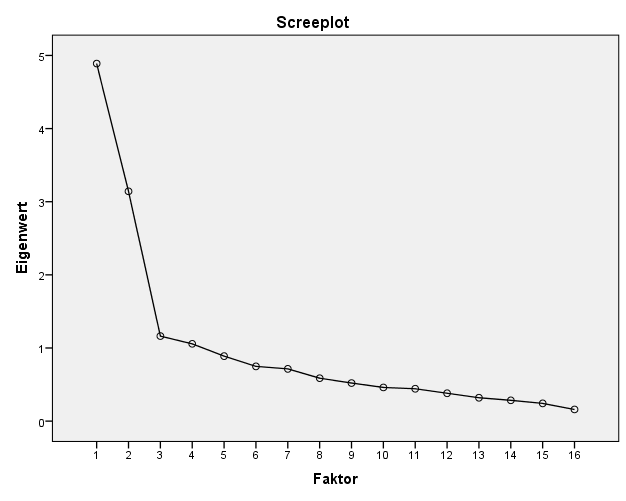


Figure: scree plot of eigenvalues of the factor analysis

Table: Additional questions after one year.

|  | **TIME POINT** |
| --- | --- |
| **QUESTION** | **after t2**  MW±SD median |
| 1. I have studied the manual after the course once again. | 3.4 ± 1.9  4.0 |
| 1. I attend the kinematics more than before the course | 5.4 ± 1.5  6.0 |
| 1. I use the ABCDE structure in the care of trauma patients. | 6.0 ± 1.2  6.0 |
| 1. My prehospital care of trauma patients has change after the course | 5.6 ± 1.4  6.0 |
| 1. After PHTLS training of the emergency medical service it is noticed a subjective change in the care of trauma patients by colleagues. | 5.9 ± 1.1  6.0 |
| 1. My prehospital care of trauma patients has improved after the course. | 5.6 ± 1.4  6.0 |
| 1. After PHTLS training of the emergency medical service it is noticed a subjective improvement in the care of trauma patients by colleagues. | 6.0 ± 0.9  6.0 |
